# Factors Influencing Clinical Reasoning Among Clinical Nurses: An Integrative Review Using the Decision‐Making Ecology Model

**DOI:** 10.1155/jonm/6956575

**Published:** 2026-08-30

**Authors:** JuHee Lee, Deokhyun Lee, Hyeran Park

**Affiliations:** ^1^ College of Nursing, Mo-Im Kim Nursing Research Institute, Yonsei University, 50-1 Yonsei-ro Seodaemun-gu, Seoul 03722, South Korea, yonsei.ac.kr; ^2^ College of Nursing, Brain Korea 21 FOUR Project, Yonsei University, 50-1 Yonsei-ro Seodaemun-gu, Seoul 03722, South Korea, yonsei.ac.kr; ^3^ Department of Nursing Science, College of Medicine, Catholic Kwandong University, 24 Beomil-ro 579beon-gil Gangneung-si, Gangwon-do 25601, South Korea, cku.ac.kr

**Keywords:** clinical decision-making, clinical judgment, clinical reasoning, critical thinking, decision-making ecology model, nurse

## Abstract

**Background:**

Clinical reasoning (CR) is a complex, context‐dependent process that is essential for safe nursing care. Existing reviews have primarily focused on nursing students rather than practicing clinical nurses. Rapid changes in patient acuity and the increasing integration of digital technologies into healthcare highlight the need for an updated synthesis that reflects contemporary clinical environments.

**Aims:**

To identify and synthesize factors influencing CR among nurses in contemporary healthcare settings using the decision‐making ecology (DME) model.

**Design:**

Integrative review.

**Methods:**

The review followed the framework proposed by Whittemore and Knafl (2005). Literature searches were conducted in CINAHL, Cochrane Library, EMBASE, and PubMed for peer‐reviewed studies published between January 2014 and June 2025. Data evaluation was performed using the Mixed Methods Appraisal Tool Version 2018.

**Results:**

A total of 25 studies were included, and 116 factors were initially extracted. After synthesis, 55 factors were identified and organized into 17 subdomains within four domains of the DME model: decision‐maker, case, organizational, and external.

**Conclusion:**

This review systematically identified multidimensional factors influencing nurses’ CR using the DME model. CR competence should be understood not solely as an individual attribute but as a capability shaped by practice, organizational, and broader sociocultural environments. The findings highlight both established determinants and emerging technology‐related influences on CR.

**Implications for Nursing Management:**

Nurse managers should provide opportunities for case‐based learning, peer feedback, and reflective discussion tailored to nurses’ levels of CR competence and clinical practice environments. Organizations should support the development of CR by ensuring adequate human and material resources, clear guidelines, and open organizational cultures that enable nurses to express and act on their clinical judgments. As technologies become more integrated into clinical practice, policymakers should promote their appropriate use to strengthen CR while minimizing technology‐related barriers.

## 1. Introduction

One of the fundamental requirements for effective nursing practice is knowing “how to think” in problem situations [1]. Clinical reasoning (CR) is the process that produces clinical judgment about patient care, and it is inherently complex, involving multiple cognitive processes [2]. CR is widely regarded as a core competency for healthcare professionals, explicitly included in Canada’s CanMEDS framework and the Tuning Educational Structures in Europe [3, 4]. The Accreditation Council for Graduate Medical Education in the United States has also emphasized CR as a core competency in its own right rather than as a subordinate concept, underscoring its growing importance [5].

Nurses, as the professionals who spend the most time at the patient’s side, play a central role in early recognition of risk factors and in making responsive decisions [6]. As patient acuity increases and patients become more informed, nurses are required to respond to more complex patient needs [5, 7, 8]. The information overload caused by the increasing use of big data analytics and artificial intelligence (AI) has also added to the complexity of nursing practice [9]. These advances underlie the importance of CR in interpreting clinical information and determining the most appropriate care [9]. Nurses with proficient CR skills can detect early signs of deterioration, anticipate risks, and initiate timely responses, thereby supporting patient safety [2, 6]. In contrast, poor CR is linked to adverse patient outcomes, such as misdiagnosis, inappropriate treatment, failure to rescue, and inadequate surveillance [5, 10, 11]. Synthesizing factors that influence nurses’ CR is necessary to support safe nursing practice and reduce preventable errors.

CR is not limited to individual abilities such as knowledge and experience. It varies according to context and is influenced by interpersonal relationships, organizational environments, and broader sociocultural conditions [11, 12]. Because these influences are embedded within dynamic healthcare systems, understanding CR requires attention to the evolving clinical environment [13]. The healthcare environment continues to evolve with the adoption of advanced technologies. Mobile devices such as personal digital assistants, smartphones, and tablets are increasingly integrated into clinical practice to support more efficient CR processes [14]. These technological and sociocultural contexts function as important factors shaping nurses’ CR [5]. Considering these rapid changes, there is a clear need for updated research on the factors influencing nurses’ CR. Identifying these multidimensional factors based on recent evidence is particularly important for nurse managers and policymakers. Such evidence can inform the development of educational, organizational, and cultural strategies that support CR in clinical practice.

Previous literature reviews on CR have primarily focused on nursing students [15, 16] and have examined CR as a learning outcome in simulation‐based education [17, 18]. Few reviews have examined the factors influencing CR among practicing clinical nurses. Existing studies include a scoping review addressing characteristics and processes of CR rather than its influencing factors [11, 19] and a review restricted to studies published before 2015 [20]. There is still a lack of comprehensive reviews that analyze factors influencing CR among clinical nurses while reflecting recent trends.

Given the context‐dependent nature of CR, it is essential to consider multidimensional factors [7, 21]. The decision‐making ecology (DME) model categorizes the influences on decision‐making into four domains: decision‐maker, case, organizational, and external [22]. The DME model was developed in child welfare to promote consistency in professional decision‐making and has been applied to studies on healthcare professionals’ reporting intentions [23]. The DME model is particularly valuable because it provides a framework for understanding influences on decision‐making at the individual level while situating it within broader contextual layers. This integrative review aimed to identify and synthesize factors influencing CR among clinical nurses using the DME model, thereby providing a structured foundation for developing strategies to strengthen CR in clinical practice.

## 2. Aims

This integrative review aimed to identify and synthesize factors influencing CR among clinical nurses using the DME model. By systematically synthesizing available evidence, this review establishes a comprehensive basis for guiding future education, organizational policy, and research aimed at strengthening nurses’ CR competence. The central research question was “Which factors influence CR among clinical nurses?”

## 3. Methods

This integrative review followed the framework proposed by Whittemore and Knafl [24], which consists of five stages: problem identification, literature search, data evaluation, data analysis, and presentation. The review was registered on the Open Science Framework (OSF) platform, and the protocol is accessible at https://doi.org/10.17605/OSF.IO/R8AZ7. The integrative review was reported in accordance with the Preferred Reporting Items for Systematic Reviews and Meta‐Analyses (PRISMA) checklist [25]. Ethics approval was not required for this integrative review because it synthesized data from previously published studies and did not involve human participants or identifiable personal data.

### 3.1. Search Strategy

The literature search was conducted in CINAHL, Cochrane Library, EMBASE, and PubMed for studies published between January 2014 and June 2025. Search strategies and terms were developed in consultation with a librarian. Combinations of relevant search terms were constructed using the Boolean operators AND and OR and applied to all database searches: (nurse∗ OR “nursing staff∗”) AND (“clinical reasoning” OR “clinical judgment” OR “clinical decision‐making” OR “critical thinking”) AND (influenc∗ OR affect∗ OR determinant∗ OR predict∗ OR association∗ OR correlation∗). Although CR is conceptually distinct from clinical judgment, clinical decision‐making, and critical thinking, the terms are often used interchangeably in the literature [5]. All four terms were included to broaden search coverage. To avoid conceptual ambiguity and maintain analytical consistency, this review uses the term CR as an inclusive term encompassing closely related constructs. The specific search terms applied to each database are detailed in Supporting A.

### 3.2. Inclusion and Exclusion Criteria

The inclusion criteria were (1) studies involving registered nurses as participants; (2) studies conducted in clinical contexts such as hospitals or other healthcare facilities; (3) original research published between January 2014 and June 2025; (4) publications in English; and (5) studies using quantitative descriptive, qualitative, or mixed‐methods research designs. The exclusion criteria were (1) studies involving nursing students, physicians, or other health professionals; (2) studies not addressing CR, clinical decision‐making, clinical judgment, or critical thinking; (3) studies that did not report influencing factors (e.g., those reporting only correlations); (4) studies focusing solely on the effect of a specific intervention on CR; and (5) non‐peer‐reviewed publications, review articles, books, case reports, commentaries, letters, and gray literature.

### 3.3. Study Selection

**FIGURE 1:**
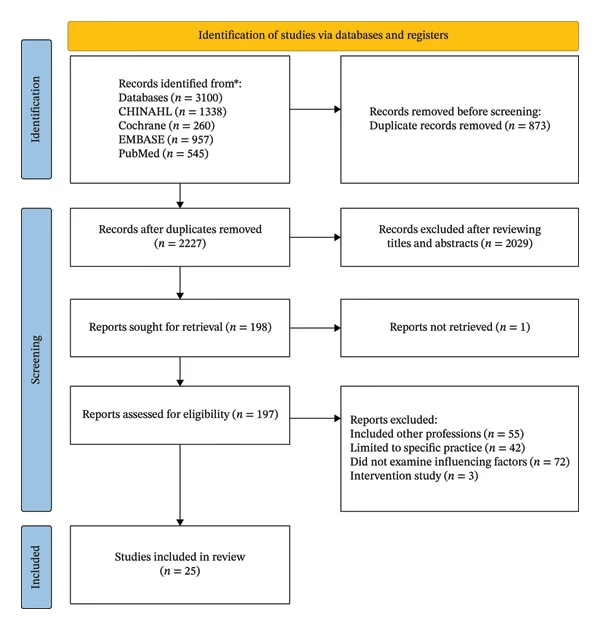
PRISMA flowchart of the study.

All search records were managed in EndNote v.21.0. The search yielded 3100 records, of which 873 duplicates were removed, leaving 2227 unique records. Two independent reviewers (D.L. and H.P.) screened titles and abstracts to assess eligibility. A total of 198 full‐text articles were retrieved and reviewed in detail by the same reviewers. During full‐text review, the reviewers discussed whether to include studies involving licensed nurses across various roles and positions. Although CR may differ depending on nursing roles, the scope of nursing practice and practice environments vary across countries and healthcare systems. Therefore, this review included all licensed nurses who provide direct or indirect nursing care to patients. This broad inclusion approach was intended to align with the aim of this review, which was to synthesize factors influencing CR across nursing practice contexts. Disagreements were resolved through discussion with the author (J.L.) until consensus was reached. In the final selection, 25 studies met the inclusion criteria. The study selection process is shown in the PRISMA flow diagram (Figure 1).

### 3.4. Quality Appraisal

Quality appraisal was conducted using the Mixed Methods Appraisal Tool (MMAT), Version 2018 [26]. The MMAT is widely used in integrative reviews because it enables evaluation of diverse study designs within a single framework. Two reviewers (D.L. and H.P.) independently assessed each study and compared their evaluations. Discrepancies were resolved through consensus. The MMAT includes two initial screening questions and five design‐specific questions. All included studies met the two screening criteria. Scores for design‐specific items ranged from 60% to 100%. In line with MMAT recommendations, no studies were excluded based on quality scores. The MMAT results are presented in Supporting B.

### 3.5. Data Extraction and Synthesis

Data extraction was conducted by one reviewer (D.L.) and checked by another reviewer (H.P.) for accuracy. Data synthesis was conducted independently by two reviewers (D.L. and H.P.), and discrepancies were resolved through discussion. When disagreements persisted, a third reviewer (J.L.) was consulted until consensus was reached.

Extracted information was organized using a structured matrix in Microsoft Excel. Each study was summarized by key variables: (a) first author, year, and country; (b) purpose; (c) study design and analysis method; (d) sample and setting; (e) measurement; and (f) findings. The structured matrix summarizing the information from the included studies is provided in Supporting C.

For quantitative descriptive studies, only statistically significant variables associated with CR were extracted. For qualitative studies, relevant expressions were identified through a line‐by‐line review of participant quotes, subthemes, and themes presented in the results.

All extracted terms were reviewed for semantic similarity and then consolidated into synthesized factors. These synthesized factors were classified into the four domains of the DME model: decision‐maker, case, organizational, and external. Several overlapping factors were also identified and categorized as cross‐domain influences. The grouping, consolidation, and classification procedures are detailed in Table 1.

**TABLE 1:** Extracted and synthesized factors influencing clinical reasoning.

Domain	Subdomain	Synthesized factor	Extracted factor	Sources
Decision‐maker	Demographics	Age	Age	[27–34]
		Gender	Sex	[33]
			Gender	[32, 35, 36]
		Educational level	Educational level	[27, 37]
			Education	[33, 38]
			Nursing degree	[39]
			Highest level of nursing education: bachelor’s degree or higher	[40]
		Marital status	Marital status	[30]
		Children status	Children status	[38]
		Religion	Religion	[12]
	Physical and psychological status	Physical status	Sick or not feeling well	[12]
		Psychological status	Under pressure and stress	[12]
			Personal circumstances (e.g., personal feelings and conditions)	[41]
			Anxiety	[28]
	Clinical experience and competence	Clinical experience	Experience	[32, 33, 37, 41]
			Total clinical experience	[31]
			Length of work history	[29]
			Years of NP experience	[34]
			Experience fosters knowledge	[6]
			Previous experience	[12]
		Self‐directed learning competence	Self‐directed learning competence (subdomain: self‐evaluation, self‐motivated belief, task analysis)	[38]
			Self‐directed learning (subdomain: planning and execution, self‐monitoring, learning motivation, interpersonal relationships)	[32]
		Practice competence	High level of practice competence	[42]
			Nurses’ ability to interpret a situation	[6]
		Communication competence	Communication competence	[31]
			Language barrier	[12]
			Emotional intelligence	[30]
	Knowledge	Academic score	Percent grade	[32]
		Knowledge	Professional knowledge	[34]
		Knowledge‐sharing attitude	Knowledge‐sharing behavior (subdomain: explicit knowledge sharing)	[43]
		Source of knowledge	Updating knowledge through various methods	[41]
			Availability of information and the presence of a more experienced nurse	[6]
	Thinking tendency	Critical thinking disposition	Critical thinking disposition (subdomain: openness and empathy, holistic and reflective disposition)	[34]
			Critical thinking disposition (subdomain: seeking truth, open‐mindedness, analytics, systematicity, self‐confidence)	[28]
			Critical‐thinking disposition	[44, 45]
		Decision‐making type	Analytic–systematic decision‐making type	[45]
			Attitude to shared decision‐making	[41]
		Leadership style	Task‐oriented conflict management leadership style	[42]
		Intuition	Intuition	[41]
			Gut feelings	[6]
		Cognitive bias	Cognitive bias	[42]
	Self‐evaluation	Core self‐evaluation	Core self‐evaluation (subdomain: locus of control, self‐esteem, self‐efficacy)	[46]
		Self‐reflection	Self‐reflection with insight	[47]
			Independent view of self	[40]
		Confidence	Confidence	[34]
			Feeling self‐confident	[42]
		Self‐efficacy	Self‐efficacy	[30]
		Self‐esteem	Self‐esteem	[40]
	Personal value	Professional attitudes and philosophies	Personal values, beliefs, and cultural awareness	[12]
			Attitude to physiology	[41]
			Attitude towards patient centeredness	[41]
			Attitude towards collaboration (e.g., constructive dialog or conflict avoidance)	[41]
		Responsibility	Keen sense of responsibility	[6]

Case	Physical factors	Physical status	Patient physical information	[41]
			Physically checking on patients	[6]
		Patient complexity	Changes in patients’ deterioration	[6]
			Complexity of patient care	[6]
			High acuity patients	[6]
	Nonphysical factors	Psychosocial status	Disadvantaged social environment, low intellectual abilities, hypochondriac, patients with anxiety disorders	[41]
			Absence of a family member	[6]
		Patient perspectives	Patients’ different traditional cultures and religious beliefs	[12]
			Preferences of patients	[41]

Case × Decision‐maker	Patient interaction stress	Patient interaction stress	Nurses’ job stressors (subdomain: patient care and interaction)	[37]
			Juggling multiple patients demands	[6]

Organizational	Work‐related conditions	Work schedule type	Shift schedule	[27]
			Work hours	[39]
		Working department	Working in an emergency unit	[33, 34, 40]
			Working unit	[33, 34]
		Position	Position	[38]
			Work position	[35]
			Professional qualification	[47]
		Monthly income	Monthly income	[38]
		Workload	Workload	[6, 12, 33, 40]
			Increased nursing workload: time pressures, heavy patient loads, lack of a nurse technician, admissions/discharges, and operational failures related to processes or missing equipment	[6]
			Workload: high patient ratio, doing non‐nursing jobs, distractions and interruptions	[12]
			Time pressures	[6]
		Staff floating system	Floating system within the organization	[12]
		Institutional condition	Quality of medical facilities, financial considerations	[41]
	Organizational systems	Technology	Utilizing technology for patient records and treatment choices	[12]
		Availability of resources	Availability of human and nonhuman (e.g., equipment and supplies) resources	[12]
			Lack of a nurse technician, missing equipment, and supplies	[6]
			Available resources	[6]
		Policy and guideline	Consistence in the application of policies and procedures and the availability of clinical guidelines	[12]
			Process failures	[6]
			Hospital protocol, linked closely to organizational structures	[42]

Organizational × Decision‐ maker	Supportive climate	Intraprofessional collaboration	Support from expert nurses	[12]
			Nursing support	[6]
			Supportive working environment	[42]
			Communication at patient handoff	[6]
			Making general agreements or discussing individual cases between maternity care professionals	[41]
		Interprofessional collaboration	Cooperation with obstetricians	[41]
			Physician conflict when calling, communicating, and engaging with physicians	[6]
			Relationship with rapid response teams	[6]
		Autonomy and empowerment	Structural empowerment	[37]
			Freedom and authority	[12, 31]
			Professional autonomy	[31]
		Compliance	Noncompliance with hospital policies and rules	[12]
			Adherence to the guidelines	[41]
		Organizational trust	Organizational Trust	[43]
	Work‐related well‐being	Coping	Positive coping	[29]
		Job burnout	Job burnout	[29]
		Occupational pressure	Occupational pressure	[29]
	Professional encouragement	Feedback	Support and correction from the nursing supervisors and hospital administration	[12]
			Continuous monitoring programs	[12]
			Perinatal audits	[41]
		Training experience	Took CDM training	[33]
			Trained nursing research in postlicensure education	[40]
			Receiving critical thinking training	[44]
			Teaching strategies during the education: PBL model	[48]
			PBL courses	[48]
			Length of exposure to PBL	[48]
			Supportive learning environment	[42]
			Nursing competency program	[12]

External	Culture	Culture	Sociocultural norms	[12]
			Legislation regarding the working conditions	[41]

External × Decision‐maker	Nomophobia	Nomophobia	Nomophobia	[49]

## 4. Results

### 4.1. General Characteristics of the Included Studies

Among the 25 included studies, two (8%) were published in 2016 and two (8%) in 2017, followed by one in 2018 (4%) and four in 2019 (16%). In both 2020 and 2021, two studies were published each year (8%), while only one was reported in 2022 (4%). A notable increase occurred in 2023, with six studies (24%), followed by a decline to four in 2024 (16%) and one in 2025 (4%). The studies were conducted across 15 countries. South Korea, China, Taiwan, Jordan, and Türkiye each contributed three studies (12%), while Ethiopia, Palestine, Iran, Japan, Croatia, Vietnam, Spain, Saudi Arabia, the Netherlands, and the United States each contributed one (4%). All studies focused exclusively on clinical nurses, involving a total of 6780 participants working in hospital settings.

Of the included studies, 21 employed a quantitative descriptive design, three used a qualitative design, and one adopted a mixed‐methods design. Twenty‐two studies that used a quantitative descriptive approach (including the quantitative results of the mixed‐methods study) measured CR scores as the dependent variable, typically based on survey data. Within these, five studies examined influencing factors by classifying CR types or strategies, and one analyzed influencing factors according to nurses’ years of clinical experience. Among the four qualitative studies (including the mixed‐methods study), one used focus group interviews, and three conducted in‐depth individual interviews to explore factors associated with CR.

### 4.2. Thematic Categorization of Influencing Factors

**FIGURE 2:**
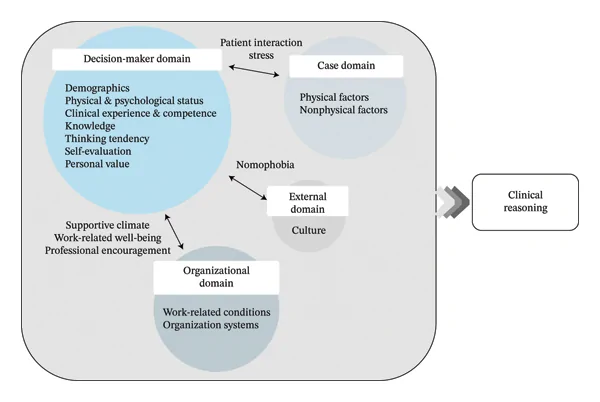
Framework of factors influencing clinical reasoning.

A total of 116 factors were initially extracted and later consolidated into 55 synthesized factors through grouping and refinement. These factors were classified into four domains according to the DME model: (1) decision‐maker, (2) case, (3) organizational, and (4) external. Seventeen subdomains were identified; of these, 12 belonged exclusively to one domain, while 5 spanned two domains, including case × decision‐maker, organizational × decision‐maker, and external × decision‐maker. The framework of categorized factors is presented in Figure 2.

#### 4.2.1. Decision‐Maker Domain

This domain included the following subdomains: demographics, physical and psychological status, clinical experience and competence, knowledge, thinking tendency, self‐evaluation, and personal values.

##### 4.2.1.1. Demographics

Higher age was associated with better CR in four studies [27–30], whereas two studies reported opposite findings [31, 32]. In addition, one study found that older nurses were more likely to use intuitive CR than analytic CR [33], while another study reported that younger nurses showed a greater tendency toward intuitive CR [34]. Higher educational level was linked to stronger CR in four studies [27, 37, 39, 40], whereas Liu et al. [38] reported the opposite association among nurses with 3–5 years of practice. One study found that nurses with a master’s degree used more intuitive CR than those with a diploma [33]. Gender was examined in four studies. One study reported higher CR among female nurses [32], while two studies found higher CR among male nurses [35, 36]. Another study reported that female nurses tended to adopt an intuitive CR approach [33]. Being married and having children were each reported to be associated with CR in one study [30, 38]. Religious affiliation was identified as a related factor in one study [12].

##### 4.2.1.2. Physical and Psychological Status

One study reported that nurses had difficulty engaging in CR when they were ill or physically unwell [12]. Negative psychological states such as stress and pressure were shown to adversely affect CR in three studies [12, 28, 41]. One study reported that anxiety levels were associated with nurses’ CR type [28].

##### 4.2.1.3. Clinical Experience and Competence

Nurses with greater clinical experience demonstrated higher levels of CR competence in eight studies [6, 12, 29, 31, 32, 34, 37, 41], while one study found that nurse practitioners with longer experience tended to use intuitive CR [33]. Self‐directed learning competence, defined as the ability to independently manage one’s own learning, was positively associated with CR in two studies [32, 38]. A high level of practice competence was associated with better CR in two studies [6, 42], and a high level of communication competence was associated with better CR in three studies [12, 30, 31].

##### 4.2.1.4. Knowledge

Higher levels of professional knowledge were positively associated with CR [34]. Higher academic scores, reflecting undergraduate academic performance, were associated with lower levels of CR [32]. One study showed that a more positive attitude toward explicit knowledge sharing, defined as a willingness to share documented information, was associated with higher levels of CR [43]. Appropriate knowledge sources such as journals, education, or experienced colleagues were identified as factors influencing the CR process in two studies [6, 41].

##### 4.2.1.5. Thinking Tendency

Critical thinking disposition was identified as a factor associated with CR in four studies [28, 34, 44, 45]. Nurses with an analytic systematic decision‐making style or a positive attitude toward shared decision‐making showed better CR [41, 45]. Intuition was identified as a contributing factor in two studies [6, 41]. One study found that nurses who exhibited higher levels of cognitive bias, and who adopted a task‐oriented approach to conflict management, demonstrated greater rational ability in CR [42].

##### 4.2.1.6. Self‐Evaluation

Core self‐evaluation, reflecting individuals’ basic beliefs about their worth and competence, was associated with higher levels of CR in one study [46]. Nurses who demonstrated better objective self‐reflection with insight exhibited higher levels of CR in two studies [40, 47]. Higher confidence was associated with intuitive CR in one study [34], and another study identified confidence as a positive factor influencing rational ability [42]. Higher levels of self‐efficacy and self‐esteem were each positively associated with CR in one study [30, 40].

##### 4.2.1.7. Personal Values

Professional attitudes and philosophies, including attitudes to physiology, patient‐centeredness, and collaboration, as well as personal values, beliefs, and cultural awareness, were identified as factors associated with CR in two studies [12, 41]. One study identified a keen sense of responsibility as a factor positively associated with CR [6].

#### 4.2.2. Case Domain

The case domain included two subdomains: physical factors and nonphysical factors. In addition, one overlapping subdomain was identified between the case and decision‐maker domains: patient interaction stress.

##### 4.2.2.1. Physical Factors

Accurate recognition and assessment of a patient’s physical conditions were identified as key elements of the CR process in two studies [6, 41]. Increasing patient complexity, particularly cases involving clinical deterioration and high‐acuity patients, was reported as a factor that hindered CR in two studies [6, 41].

##### 4.2.2.2. Nonphysical Factors

Psychosocial factors, including psychological vulnerability and socially disadvantaged family background, were identified as important influences in two studies [6, 41]. Patient‐related perspectives, such as preferences and beliefs shaped by cultural or religious values, were also identified as factors influencing CR in two studies [12, 41].

#### 4.2.3. Interaction Between the Case and Decision‐Maker Domains

##### 4.2.3.1. Patient Interaction Stress

Job stress related to patient care and communication [37] and the burden of managing patient demands [6] showed negative associations with nurses’ CR.

#### 4.2.4. Organizational Domain

The organizational domain included two subdomains: work‐related conditions and organizational systems. Overlapping subdomains between the organizational and decision‐maker domains included supportive climate, work‐related well‐being, and professional encouragement.

##### 4.2.4.1. Work‐Related Conditions

Work schedule type was examined in two studies. Nurses working fixed 7‐h shifts demonstrated stronger CR compared with those working 12‐h day or night shifts [27]. In another study, work hours were positively associated with CR [39]. The working department was identified as an influencing factor in three studies, each comparing different units: intensive care unit > outpatient department [33]; internal medicine > critical care, obstetrics, pediatrics, psychiatric [34]; and emergency unit > other departments [40]. Position was also identified in three studies: a higher position was associated with better CR [38, 47], with nurse managers > general nurses and administrative assistants [35]. Higher monthly income was associated with higher levels of CR in one study [38]. Workload was identified in four studies, with inconsistent findings. In two of these, heavier workload was associated with lower levels of CR competence [6, 12], whereas one study reported the opposite association [40]. In another study, nurses with higher workload were more likely to use intuitive CR [33]. Frequent staff floating systems were reported to inhibit CR by exposing nurses to unfamiliar clinical situations [12]. Institutional facilities and financial resources were identified as factors influencing CR in one study [41].

##### 4.2.4.2. Organizational Systems

Appropriate use of technology was identified as a factor positively influencing CR in one study [12]. Availability of both human and nonhuman resources supported CR in two studies [6, 12]. The presence of clear, systematic policies and guidelines was identified as a positive influence on CR in three studies [6, 12, 42].

#### 4.2.5. Interaction Between the Organizational and Decision‐Maker Domains

##### 4.2.5.1. Supportive Climate

Intraprofessional collaboration, including supportive environments and effective communication among nurses, was identified as a positive influence in four studies [6, 12, 41, 42]. Interprofessional collaboration, including relationships with physicians and rapid response teams, was highlighted in two studies [6, 41]. Organizational autonomy and empowerment were identified in three studies as positively associated with nurses’ CR [12, 31, 37]. Compliance with predetermined guidelines was positively associated with nurses’ CR in two studies [12, 41]. One study reported that greater trust in the organization was positively associated with CR [43].

##### 4.2.5.2. Work‐Related Well‐Being

Higher levels of positive coping and lower levels of job burnout and occupational stress were associated with stronger CR in one study [29].

##### 4.2.5.3. Professional Encouragement

Supervisor or institutional feedback, including continuous monitoring and audit, was identified as a positive influence in two studies [12, 41]. Training experience was assessed in six studies; four reported that continuous and supportive training was positively associated with CR [12, 31, 40, 48]. Two studies that examined CR types as outcomes reported mixed results, as one study reported a positive and the other reported a negative association with intuitive CR [33, 44].

#### 4.2.6. External Domain

The external domain included one subdomain (culture) and one overlapping subdomain with the decision‐maker domain (nomophobia).

##### 4.2.6.1. Culture

Culture was identified in two studies, which reported sociocultural norms and legislation regarding working conditions as factors influencing CR [12, 41].

#### 4.2.7. Interaction Between the External and Decision‐Maker Domains

##### 4.2.7.1. Nomophobia

Nomophobia, defined as fear or anxiety associated with being without a mobile phone, was negatively associated with CR competence in one study [49].

## 5. Discussion

This integrative review identified multiple factors influencing nurses’ CR, organized into the decision‐maker, case, organizational, and external domains. The findings highlight that CR is a multidimensional construct shaped by both personal characteristics and broader contextual conditions. By systematically categorizing these influences, this review provides a foundation for future educational, policy, and research initiatives aimed at strengthening CR among nurses.

The decision‐maker domain yielded the largest number of influencing factors. These results align with earlier reviews that identified individual attributes such as age, educational level, clinical experience, intuition, thinking tendency, and confidence as critical elements of clinical nurses’ decision‐making [19, 20]. These attributes have been regarded as central determinants of CR since the 1980s, when research on CR first began to emerge [7, 50–52]. Recent studies continue to confirm their importance in defining the characteristics of nurses’ CR [11]. Although healthcare environments have changed substantially over the past four decades, these findings suggest that individual attributes remain essential to effective CR. To address the modifiable factors in the decision‐maker domain, organizations should provide educational opportunities tailored to nurses’ levels of CR competence to support progressive development over time [11, 53].

The case domain reflected the inherent complexity and variability of patients and was divided into physical and nonphysical factors. Nonphysical factors included contextual aspects such as personal beliefs shaped by religion or culture and the influence of family members [6, 12, 41]. These findings are consistent with prior reviews that emphasized the importance of gathering detailed and diverse information about the patient in CR [5, 11, 54]. Case‐based simulation education has been reported to enhance nurses’ CR by enabling them to practice integrating complex patient‐related contextual factors [55]. Hsieh et al. [56] demonstrated that scenario‐based simulation incorporating patients’ religious beliefs, spiritual concerns, and family support significantly improved nurses’ competence in responding to patients’ needs. In clinical practice where complex and diverse patient cases are encountered, practical strategies may include creating opportunities to share specific cases with colleagues and engage in peer feedback and reflection [57, 58].

At the intersection of the case and decision‐maker domains, patient interaction stress has been identified as a negative influencing factor [6, 37]. This finding is consistent with earlier research that identified poor interactions as barriers to high‐quality care [59, 60]. Challenges in nurse–patient interactions are not solely attributable to individual communication skills or language proficiency [59, 60]. They are shaped by institutional conditions because heavy workloads and staffing shortages reduce opportunities for patient interactions and may shift communication toward task‐centered rather than person‐centered communication [59]. These findings suggest that task‐management strategies, together with education to strengthen communication skills, may help reduce patient interaction stress and support CR.

The organizational domain captured the influence of work‐related conditions and organizational systems. Three recurring organizational conditions shaped nurses’ CR. First, inadequate workforce management, including long shifts, heavy workloads, and frequent staff floating, was repeatedly identified as a barrier to CR. These conditions expose nurses to time pressure and unfamiliar clinical situations, thereby undermining the conditions necessary for effective CR. Second, the availability of material resources, including hospital infrastructure, diagnostic equipment, and information technology needed for treatment, influenced nurses’ CR. Inadequate organizational support may limit access to essential information and increase the risk of reasoning errors [54]. Third, clear and consistently implemented guidelines supported CR by reducing uncertainty, promoting practice consistency, and helping nurses make evidence‐informed decisions in complex situations. To support CR, organizations should ensure adequate human and material resources, monitor workload, and provide clear clinical guidelines [5, 11, 61].

At the intersection of the organizational and decision‐maker domains, supportive climates, work‐related well‐being, and professional encouragement were found to enhance CR. A closed organizational culture may prevent nurses from translating sound clinical judgments into action, even when appropriate reasoning has occurred [62, 63]. Nurse managers should, therefore, cultivate an open organizational culture in which nurses can communicate concerns freely, act on their clinical judgments, and receive constructive feedback that supports effective CR [64].

The external domain identified cultural influences, including sociocultural norms and legal frameworks, as determinants of reasoning processes [12, 41]. Culture shapes both the content and style of thinking that support CR [65]. Nurses’ cultural backgrounds and sociocultural norms are deeply rooted and may not be easily changed. Nurse managers should, therefore, provide education and reflective discussion opportunities that help nurses recognize how these factors may influence their CR. Such reflection can help nurses critically examine their judgments and translate this awareness into more patient‐centered clinical practice [66].

A newly identified factor was nomophobia, defined as anxiety about being without a mobile phone, which was shown to negatively influence CR [49]. These findings are consistent with previous cross‐sectional studies reporting that nomophobia is common among nurses and nursing students and may adversely affect concentration [67, 68]. A recent systematic review reported that 68.15% of the participants experienced at least some degree of nomophobia [68, 69]. Although some studies suggest that mobile devices can support CR, evidence explaining how nomophobia affects CR remains limited [70]. Further research is needed to inform policies and educational strategies for appropriate mobile phone use in clinical settings.

Several personal and organizational factors identified in this review have also been frequently reported in previous reviews of nurses’ job satisfaction or job retention [71, 72]. This review, by focusing on CR as a complex cognitive process, encompassed a broader range of influencing factors, including patients’ complex needs, cognitive characteristics, and technology‐related factors. This review contributes to a comprehensive understanding of nurses’ CR by integrating these multidimensional influences within the DME model.

By synthesizing recent evidence to reflect contemporary clinical contexts, this review identified established and newly emerging factors affecting nurses’ CR. The findings emphasize that nurses’ CR cannot be explained solely by individual attributes but rather emerges from the continuous interactions among individual, patient, organizational, and external domains. These domains are interdependent, and recognizing their dynamic interplay is crucial for designing educational interventions and guiding organizational strategies. Technology was identified as an emerging factor influencing CR, highlighting the importance of organizational preparedness as AI and robotics become increasingly integrated into clinical practice.

## 6. Implications for Nursing Management

First, grounded in the DME model, this review provides a structured framework for designing educational interventions and organizational strategies that address the multidimensional influences on nurses’ CR. Nurse managers should draw on this framework to provide learning opportunities through complex case reviews, peer interaction, and reflective discussion.

Second, the findings underscore the importance of organizational support, including effective workforce management, adequate material resources, and clear, consistently applied guidelines. Organizations should also foster open cultures that enable nurses to express concerns and act on their clinical judgments.

Third, the identification of nomophobia as an emerging barrier to CR indicates the need for proactive policy responses. Policymakers should develop clear guidelines for mobile device use in clinical settings and provide digital well‐being education to promote appropriate technology use without disrupting CR.

## 7. Limitations of the Study

Several limitations should be considered when interpreting the findings of this integrative review. First, although a comprehensive search was conducted across multiple databases, only studies published in English were included. This language restriction may have excluded relevant evidence published in other languages. Second, this review focused on general factors influencing nurses’ CR and judgment, without addressing highly specific contexts such as restraint use or triage decision‐making. Future research that considers such context‐specific influences may identify additional factors shaping CR. Third, heterogeneity in study settings and measurement tools increased the complexity of synthesizing findings, requiring cautious interpretation of the results. Finally, this review systematically identified factors influencing CR across the DME domains; however, interactions among decision‐maker, case, organizational, and external factors remain insufficiently understood. Future research should further examine how these factors interact to influence CR competence across diverse clinical settings.

## 8. Conclusion

This integrative review synthesized factors influencing CR among clinical nurses using the DME model and identified 55 factors across the decision‐maker, case, organizational, and external domains. The findings indicate that CR competence is shaped by the dynamic interplay of multidimensional contextual factors rather than individual attributes alone. Strengthening CR, therefore, requires multifaceted strategies, including case sharing, peer feedback, workload‐monitoring systems, and open organizational communication cultures. Technology‐related factors, including nomophobia, emerged as newly identified determinants, underscoring the need for further research to clarify how diverse technologies may support or hinder effective CR in clinical practice.

## Author Contributions

Study design: JuHee Lee, Deokhyun Lee, and Hyeran Park.

Formal analysis: Deokhyun Lee and Hyeran Park.

Validation: JuHee Lee, Deokhyun Lee, and Hyeran Park.

Study supervision: JuHee Lee.

Writing–original draft: Deokhyun Lee and Hyeran Park.

Writing–review and editing: JuHee Lee, Deokhyun Lee, and Hyeran Park.

## Funding

This research was supported by Yonsei “Eokkaedongmu Project” through the 4th BK21 Graduate School Innovation Support Project funded by the Ministry of Education. This work was supported by the National Research Foundation of Korea (NRF) grant funded by the Korea government (MSIT) (RS‐2026‐25476136). The author Deokhyun Lee received a scholarship from Brain Korea 21 FOUR Project funded by National Research Foundation (NRF) of Korea, Yonsei University College of Nursing.

## Disclosure

Protocol Registration: Open Science Framework (https://doi.org/10.17605/OSF.IO/R8AZ7). All authors have read and approved the final manuscript.

## Conflicts of Interest

The authors declare no conflicts of interest.

## Supporting Information

Additional supporting information can be found online in the Supporting Information section.

## Supporting information


**Supporting Information 1** The supporting files include Supporting A (search terms), Supporting B (quality appraisal of included studies using MMAT), and Supporting C (summary of included studies).

**Supporting Information 2** PRISMA Checklist.

## Data Availability

Extracted data are available in the article and supporting files. Additional extraction materials are available from the corresponding authors upon reasonable request.
