# Supplementary material for: Factors Influencing Clinical Reasoning Among Clinical Nurses: An Integrative Review Using the Decision‐Making Ecology Model
Source: J Nurs Manag. 2026 Aug 30;2026:6956575. doi: 10.1155/jonm/6956575 (PMC13526239; doi:10.1155/jonm/6956575)
Supplement: Supplementary file 2 [file JONM-2026-6956575-s002.zip › Supplementary A.docx]

[Supplementary A. Search terms]

| **Database** | **Search terms** |
| --- | --- |
| CINAHL | S1) (MH "Nurses+") OR (MH "Nursing Staff, Hospital") OR (MH "Staff Nurses") S2) TI (nurse OR "nursing staff*") OR AB (nurse OR "nursing staff*") S3) S1 OR S2 S4) (MH "Clinical Reasoning") OR (MH "Decision Making, Clinical") OR (MH "Critical Thinking") S5) TI ("clinical reasoning" OR "clinical judgment" OR "clinical decision making" OR "clinical decision-making" OR "critical thinking") OR AB ("clinical reasoning" OR "clinical judgment" OR "clinical decision making" OR "clinical decision-making" OR "critical thinking") S6) S4 OR S5 S7) TI (influenc* OR affect* OR determinant* OR predict* OR association* OR correlation*) OR AB (influenc* OR affect* OR determinant* OR predict* OR association* OR correlation*) S8) S3 AND S6 AND S7 |
| Cochrane | #1) [mh "Nurses"] OR [mh "Nursing Staff"] #2) nurse:ti,ab,kw OR (nursing NEXT staff*):ti,ab,kw #3) #1 OR #2 #4) [mh "Clinical Reasoning"] OR [mh "Clinical Decision-Making"] #5) (clinical reasoning):ti,ab,kw OR (clinical judgment):ti,ab,kw OR (clinical decision making):ti,ab,kw OR (critical thinking):ti,ab,kw #6) #4 OR #5 #7) influenc*:ti,ab,kw OR affect*:ti,ab,kw OR determinant*:ti,ab,kw OR predict*:ti,ab,kw OR association*:ti,ab,kw OR correlation*:ti,ab,kw #8) #3 AND #6 AND #7 |
| EMBASE | #1) 'nurse'/exp OR 'nursing staff'/exp #2) nurse:ab,ti OR 'nursing staff*':ab,ti #3) #1 OR #2 #4) 'clinical reasoning'/exp OR 'clinical decision making'/exp OR 'critical thinking'/exp #5) 'clinical reasoning':ab,ti OR 'clinical judgment':ab,ti OR 'clinical decision making':ab,ti OR 'clinical decision-making':ab,ti OR 'critical thinking':ab,ti #6) #4 OR #5 #7) influenc*:ab,ti OR affect*:ab,ti OR determinant*:ab,ti OR predict*:ab,ti OR association*:ab,ti OR correlation*:ab,ti #8) #3 AND #6 AND #7 |
| PubMed | #1) "nurses"[MeSH Terms] OR "nursing staff"[MeSH Terms] #2) nurse [Title/Abstract] OR "nursing staff*"[Title/Abstract] #3) #1 OR #2 #4) "Clinical Reasoning"[MeSH Terms] OR "Clinical Decision-Making"[MeSH Terms] #5) "clinical reasoning"[Title/Abstract] OR "clinical judgment"[Title/Abstract] OR "clinical decision making"[Title/Abstract] OR "clinical decision-making"[Title/Abstract] OR "critical thinking"[Title/Abstract] #6) #4 OR #5 #7) influenc*[Title/Abstract] OR affect*[Title/Abstract] OR determinant*[Title/Abstract] OR predict*[Title/Abstract] OR association*[Title/Abstract] OR correlation*[Title/Abstract] #8) #3 AND #6 AND #7 |
