# Supplementary material for: Factors Influencing Clinical Reasoning Among Clinical Nurses: An Integrative Review Using the Decision‐Making Ecology Model
Source: J Nurs Manag. 2026 Aug 30;2026:6956575. doi: 10.1155/jonm/6956575 (PMC13526239; doi:10.1155/jonm/6956575)
Supplement: Supplementary file 2 [file JONM-2026-6956575-s002.zip › Supplementary B.docx]

[Supplementary B. Quality Appraisal of Included Studies Using MMAT]

|  | S1. Are there clear research questions? | S2. Do the collected data allow to address the research questions? | 1.1. Is the qualitative approach appropriate to answer the research question? | 1.2. Are the qualitative data collection methods adequate to address the research question? | 1.3. Are the findings adequately derived from the data? | 1.4. Is the interpretation of results sufficiently substantiated by data? | 1.5. Is there coherence between qualitative data sources, collection, analysis and interpretation? | 4.1 Is the sampling strategy relevant to address the research question? | 4.2 Is the sample representative of the target population? | 4.3 Are the measurements appropriate? | 4.4 Is the risk of nonresponse bias low? | 4.5 Is the statistical analysis appropriate to answer the research question? | 5.1. Is there an adequate rationale for using a mixed methods design to address the research question? | 5.2. Are the different components of the study effectively integrated to answer the research question? | 5.3. Are the outputs of the integration of qualitative and quantitative components adequately interpreted? | 5.4. Are divergences and inconsistencies between quantitative and qualitative results adequately addressed? | 5.5. Do the different components of the study adhere to the quality criteria of each tradition of the methods involved? |
| --- | --- | --- | --- | --- | --- | --- | --- | --- | --- | --- | --- | --- | --- | --- | --- | --- | --- |
| First Author, Year | Screening  questions | | Qualitative | | | | | Quantitative descriptive | | | | | Mixed methods | | | | |
| Abu Arra, 2023 | Y | Y |  |  |  |  |  | Y | Y | Y | Y | Y |  |  |  |  |  |
| Abate, 2022 | Y | Y |  |  |  |  |  | Y | Y | Y | Y | Y |  |  |  |  |  |
| AL Btoush, 2025 | Y | Y |  |  |  |  |  | N | Y | Y | Y | Y |  |  |  |  |  |
| Ali-Abadi, 2020 | Y | Y |  |  |  |  |  | Y | Y | Y | Y | Y |  |  |  |  |  |
| Chen, 2016 | Y | Y |  |  |  |  |  | N | Y | Y | Y | Y |  |  |  |  |  |
| Chen, 2019 | Y | Y |  |  |  |  |  | Y | Y | Y | Y | Y |  |  |  |  |  |
| Farčić, 2020 | Y | Y |  |  |  |  |  | N | Y | Y | Y | N |  |  |  |  |  |
| Futami, 2019 | Y | Y |  |  |  |  |  | N | Y | Y | Y | Y |  |  |  |  |  |
| Gao, 2023 | Y | Y |  |  |  |  |  | N | Y | Y | Y | Y |  |  |  |  |  |
| Kapikiran, 2023 | Y | Y |  |  |  |  |  | N | Y | Y | Y | Y |  |  |  |  |  |
| Kaplan, 2024 | Y | Y |  |  |  |  |  | Y | Y | Y | Y | CT |  |  |  |  |  |
| Liu, 2024 | Y | Y |  |  |  |  |  | N | Y | Y | Y | Y |  |  |  |  |  |
| Noh, 2024 | Y | Y |  |  |  |  |  | N | Y | Y | Y | Y |  |  |  |  |  |
| Park, 2023 | Y | Y |  |  |  |  |  | N | Y | Y | Y | Y |  |  |  |  |  |
| Polat, 2019 | Y | Y |  |  |  |  |  | Y | Y | Y | Y | Y |  |  |  |  |  |
| Rababah, 2024 | Y | Y |  |  |  |  |  | N | Y | Y | Y | Y |  |  |  |  |  |
| Van Nguyen, 2021 | Y | Y |  |  |  |  |  | N | Y | Y | Y | CT |  |  |  |  |  |
| Wu, 2016 | Y | Y |  |  |  |  |  | N | Y | Y | Y | Y |  |  |  |  |  |
| Wu, 2023 | Y | Y |  |  |  |  |  | N | Y | Y | Y | Y |  |  |  |  |  |
| Yoo, 2019 | Y | Y |  |  |  |  |  | Y | Y | Y | Y | Y |  |  |  |  |  |
| Zuriguel-Pérez, 2018 | Y | Y |  |  |  |  |  | N | Y | Y | Y | Y |  |  |  |  |  |
| Alaseeri, 2021 | Y | Y | Y | Y | Y | Y | Y |  |  |  |  |  |  |  |  |  |  |
| Daemers, 2017 | Y | Y | Y | Y | Y | Y | Y |  |  |  |  |  |  |  |  |  |  |
| Dresser, 2023 | Y | Y | Y | Y | Y | Y | Y |  |  |  |  |  |  |  |  |  |  |
| Lean Keng, 2017 | Y | Y |  |  |  |  |  |  |  |  |  |  | Y | Y | Y | Y | N |
