# Supplementary material for: Factors Influencing Clinical Reasoning Among Clinical Nurses: An Integrative Review Using the Decision‐Making Ecology Model
Source: J Nurs Manag. 2026 Aug 30;2026:6956575. doi: 10.1155/jonm/6956575 (PMC13526239; doi:10.1155/jonm/6956575)
Supplement: Supplementary file 2 [file JONM-2026-6956575-s002.zip › Supplementary C.docx]

[Supplementary C. Summary of Included Studies]

| No | 1^st^ Author, Year, Country | Purpose | Study design,  Analysis method | Participants (sample size) Setting | Measurement | Findings |
| --- | --- | --- | --- | --- | --- | --- |
| 1 | Abu Arra, 2023, Palestine | To assess the factors influencing nurses’ CDM in the emergency department. | Cross-sectional study, multiple linear regression analysis | ER RNs (n=227)  from government hospitals | Clinical Decision-Making in Nursing Scale | Nursing degree (↑, *b* = 0.194, *p* = 0.001) Work hours (↑, *b* = 0.090, *p* = 0.003) |
| 2 | Abate, 2022, Ethiopia | To assess the CDM approaches (analytical vs. intuitive) among nurses and to identify personal and work-related factors associated with using an intuitive decision-making approach. | Cross-sectional survey, logistic regression analysis | RNs (n = 393) from university hospital | Decision-making  Approach Instruments  (analytical/ intuitive) | aOR reflects the likelihood of being in the intuitive group compared to those using analytic group.  Age ≥ 34 (↑, aOR = 3.98, *p* = 0.001) (ref: 24-28) Sex: `2Female (↑, aOR = 2.07, *p* = 0.002) (ref: male) Working unit: ICU (↑, aOR = 3.06, *p* = 0.019) (ref: OPD) Education level: Master’s degree (↑, aOR = 3.49, *p* = 0.013) (ref: Diploma) Experience >10 years (↑, aOR = 3.19, *p* = 0.001) (ref: **≤** 5 years) Workload (↓, aOR = 0.54, *p* = 0.024) (ref: high) CDM training taken (↑, aOR = 3.11, *p* = 0.001) (ref: no) |
| 3 | AL Btoush, 2025, Jordan | To assess the relationship between emotional intelligence, self-efficacy, and CDM among critical care nurses. | Cross-sectional survey, partial least squares path analysis | Critical care unit RNs (n = 407) from government, private, and university hospitals | Clinical Decision-Making in Nursing Scale | Age (↑, β = 0.182, *p* = .01) Emotional intelligence (↑, β = 0.218, *p* < .001) Marital status (↑, β = 0.092, *p* < .05) (ref. single) Self-efficacy (↑, β = 0.328, *p* < .001) |
| 4 | Ali-Abadi, 2020, Iran | Comparing the level of critical thinking skills in ICU and medical-surgical nurses and identifying factors explaining these skills. | Cross-sectional survey, multiple linear regression analysis | ICU RNs (n = 60) Medical-surgical RNs (n = 60)  from university hospital | California Critical Thinking Skills Test – Form B | Gender (female ↓, *b*= -0.29, *p* = 0.001) |
| 5 | Chen, 2016, Taiwan | To investigate nurse practitioners’ CDM abilities and to identify factors associated with their analytical and intuitive decision-making styles. | Cross-sectional survey, hierarchical multiple regression analysis | Nurse practitioner (n = 197) from one medical center and its four healthcare system hospitals | Clinical Decision-Making Model Inventory (analytical/intuitive) | **Analytic Decision Making:** Years of NP experience (↑, β = 0.21, *p* < .01) Working unit: Critical care (↓, β = -0.14, *p* < .05) (ref. internal medicine) Professional knowledge (↑, β = 0.36, *p* < .01) CTD_Openness and empathy (↑, β = 0.21, *p* < .01) CTD_Holistic and reflective disposition (↑, β = 0.22, *p* < .05) **Intuitive Decision Making:**  Age (↓, β = -0.15, *p* < .05) Years of NP experience (↑, β = 0.21, *p* < .01) Working unit: Obstetrics/Pediatrics/Psychiatric (↓, β = -0.20, *p* < .01) (ref. internal medicine) Working unit: Critical care (↓, β = -0.21, *p* < .01) (ref. internal medicine) Professional knowledge (↑, β = 0.32, *p* < .05) Confidence (↑, β = 0.17, *p* < .05) CTD_Holistic & reflective disposition (↑, β = 0.30, *p* < .01) |
| 6 | Chen, 2019, Taiwan | To compare self-reflection and critical thinking levels between novice and experienced registered nurses, and to examine how professional qualifications and self-reflection with insight influence CTD in experienced nurses. | Cross-sectional survey, partial least squares  structural equation modeling | Novice RNs n = 297 Experienced RNs n = 300  from one medical center | Taiwan Critical Thinking Disposition Inventory | Self-reflection with insight (↑, β = 0.24, *p* < .001) Professional qualifications (i.e. age, years of job experience, and position on the clinical ladder) (↑, β = 0.18, *p* < .001) |
| 7 | Farčić, 2020, Croatia | To examine whether core self-evaluation personality traits (self-esteem, self-efficacy, locus of control, emotional stability) predict CDM among nurses. | Cross-sectional survey hierarchical multiple regression | RNs (n = 584) from university hospital | Clinical Decision Making in Nursing Scale | Core self-evaluation_Locus of control (↑, β = 0.356, *p* < .005) Core self-evaluation_Self-esteem (↑, β = 0.460, *p* < .005) Core self-evaluation_Self-efficacy (↑, β = 0.264, *p* < .005) |
| 8 | ^*^Futami, 2020, Japan | To comprehensively examine which organizational and personal factors are associated with hospital nurses’ CTD in Japan | Cross-sectional survey, multiple regression analysis | Staff nurses (n = 986) from 61 hospitals | Japanese Critical Thinking Disposition Scale | Working in an emergency unit (↑, β = 0.07, *p* = .040) Highest level of nursing education: bachelor’s degree or higher (↑, β = 0.07, *p* = .030) Independent view of self (↑, β = 0.22, *p* < .001) Self-esteem (↑, β = 0.24, *p* < .001) Workload (↑, β = 0.10, *p* = .002) Trained nursing research in post-licensure education (↑, β = 0.15, *p* < .001) |
| 9 | Gao, 2023, China | To determine how job burnout affects CDM consciousness and stress coping styles among nurses | Cross-sectional survey, multiple linear regression analysis | RNs (n = 277) from university hospital | Clinical Decision-Making in Nursing Scale | Positive coping (↑, β = 0.260, *p* < .001) Job burnout (↓, β = –0.272, *p* = .004) Occupational pressure (↓, β = –0.053, *p* = .045) Age (↑, β = 0.040, *p* = .021) Length of work history (↑, β = 0.109, *p* = .020) |
| 10 | Kapikiran, 2023, Türkiye | To examine how nomophobic behaviors affect CDM perceptions among emergency room nurses. | Cross-sectional survey, simple linear regression analysis | ER RNs (n = 186) from four hospitals | Clinical Decision-Making in Nursing Scale | Nomophobia (↓, β = –0.730, *p* < .001) |
| 11 | Kaplan, 2024, Türkiye | To determine the levels of rational and intuitive decision-making styles and CTD of nurses and to identify how critical thinking affects both decision-making styles. | Cross-sectional survey, multiple linear regression analysis | RNs (n = 355) from two tertiary hospitals | Decision-making Styles (rational/ intuitive) Scale | **Rational decision-making style:** Critical Thinking Disposition (↑, β = 0.707, *p* < .001)  **Intuitive decision-making style:**  Critical thinking disposition (↑, β = 0.371, *p* < .001) Receiving critical thinking training (↓, β = –0.147, *p* = .003) |
| 12 | Liu, 2024, China | To compare differences in clinical reasoning competence among nurses with different working years, examine their relationship with self-directed learning competence, and identify how self-directed learning competence predicts clinical reasoning competences. | Cross-sectional survey, multivariate regression analysis | RNs (n = 376)  from four general hospitals | Nurses’ Clinical Reasoning Scale | **<1 year:**  Self-motivated belief (↑, β = 0.602, *p* < .001) **≥1 year to <3 years:**  Monthly income (↑, β = 0.133, *p* < .05) Self-directed learning competence_Self-motivated belief (↑, β = 0.368, *p* < .001) Self-directed learning competence_Task analysis (↑, β = 0.335, *p* < .001) **3–5 years:** Position (↑, β = 0.245, *p* < .05) Monthly income (↑, β = 0.256, *p* < .05) Education (↓, β = –0.344, *p* < .05) Children status (↑, β = 0.226, *p* < .05)  Self-directed learning competence_Self-motivated belief (↑, β =0.579, *p* < .001) **6–10 years:** Self-directed learning competence_Self-motivated belief (↑, β = 0.744, *p* < .001) **10 years:**  Monthly income (↑, β = 0.256, *p* < .05) Self-directed learning competence_Self-evaluation (↑, β = 0.280, *p* < .05) Self-directed learning competence_Self-motivated belief (↑, β = 0.523, *p* < .001) |
| 13 | Noh, 2024, South Korea | To investigate the relationships among communication competence, professional autonomy, and clinical reasoning competence in oncology nurses, and to identify the key predictors of clinical reasoning competence for enhancing reasoning performance in cancer nursing practice. | Cross-sectional survey, hierarchical multiple regression analysis | Oncology RNs (n = 147) from secondary or tertiary hospitals | Nurses’ Clinical Reasoning Scale | Age (↓, β = –0.31, *p* = 0.002) Total clinical experience (↑, β = 0.48, *p* < .001) Communication competence (↑, β = 0.44, *p* < .001) Professional autonomy (↑, β = 0.27, *p* < .001) |
| 14 | Park, 2023, South Korea | To identify factors influencing the decision-making ability of clinical nurses, to examine the structural relationships among influencing variables. | Cross-sectional survey, path analysis | RNs (n = 274) from two general hospitals | Decision-making ability scale | Analytic-systematic decision-making type (direct, β = 0.401, *p* < .01) Critical thinking disposition (direct, β = 0.261, *p* < .01) |
| 15 | Polat, 2019, Türkiye | To examine the relationships between nurses’ state-trait anxiety, CTD, and DMS. | Cross-sectional survey, multiple linear regression | RNs (n = 326) from university hospitals | Decision-Making Strategies Scale | **Independent DMS:** Age (↑, *b* = 0.079, *p* = .001) CTD_Systematicity (↑, *b* = 0.231, *p* < .001)  CTD_Self-confidence (↑, *b* = 0.098, *p* = .020) Anxiety level (↓, *b* = –0.057, *p* < .001) **Intuitive DMS:** CTD_Seeking truth (↓*, b* = –0.101, *p* = .041) CTD_Open-mindedness (↓, *b* = –0.144, *p* < .001) Age (↑, *b* = 0.052, *p* = .027) **Rational DMS:** CTD_Open-mindedness (↓, *b* = –0.081, *p* = .003) CTD_Analytics (↑, *b* = 0.237, *p* < .001) CTD_Systematicity (↑, *b* = 0.114, *p* = .044) Age (↑, *b* = 0.049, *p* = .019) **Indecision strategy:** CTD_Open-mindedness (↓, *b* = –0.148, *p* < .001)  CTD_Systematicity (↓, *b* = –0.239, *p* < .001) CTD_Self-confidence (↓, *b* = –0.084, *p* = .043) Anxiety level (↑, *b* = 0.088, *p* < .001) |
| 16 | Rababah, 2024, Jordan | To investigate potential predictors of CDM among registered nurses, focusing on self-directed learning dimensions and demographic characteristics, to provide evidence for interventions that strengthen nurses’ decision-making abilities. | Cross-sectional survey, hierarchical multiple regression analysis | RNs (n = 139) from main referral hospital | Decision-making instrument-revised | Age (↓, β = –0.26, *p* = 0.005) Percent grade (↓, β = –0.38, *p* < .001) Experience (↑, β = 0.33, *p* = 0.005) Self-directed learning_Learning Motivation (↓, β = –0.42, *p* < .001) Self-directed learning_Planning and Execution (↑, β = 0.36, *p* = 0.002) Self-directed learning_Self-Monitoring (↑, β = 0.41, *p* = 0.007) Self-directed learning_Interpersonal Relationships (↓, β = –0.34, *p* = 0.027) Gender (↑, β = 0.38, *p* = 0.036) (ref: male) |
| 17 | Van Nguyen, 2021, Vietnam | To examine the level of critical thinking of professional nurses and to explore the work-related and personal-related factors associated with it, providing baseline data to inform strategies for improving nurses' critical thinking ability. | Cross-sectional survey, stepwise multiple regression | Professional nurses (n = 420) from three major public hospitals | Nursing Critical Thinking in Clinical Practice Questionnaire | Work position: Administrative assistant (↓, β = –0.351, *p* < .001) (ref: nurse manager) Work position: General nurse (↓, β = –0.330, *p* < .001) (ref: nurse manager) Gender: Female (↓, β = –0.098, *p* = .037) (ref: male) |
| 18 | Wu, 2016, China | To identify factors influencing Chinese nurses’ CDM skills using quantile regression | Cross-sectional survey, quantile regression and ordinary least squares regression | RNs (n = 216) from two university hospitals | Practical Knowledge Inventory for Nurses | Experience (↑, β = .22, *p* < .05) Educational level (↑, β = .30, *p* < .01) Perceived nurse–patient relationship (↓, β = –.16, *p* < .01) Total structural empowerment (↑, β = .24, *p* < .05) Nurses’ job stressors _Patient care and interaction (↓, β = –.41, *p* < .01) |
| 19 | Wu, 2023, Taiwan | To describe the level of CTD among new graduate nurses and to examine factors influencing its development, providing evidence to strengthen CTD through appropriate educational strategies. | Cross-sectional survey, multiple linear regression | New graduate nurses (n = 100)  from two regional teaching hospitals | California Critical Thinking Disposition Inventory | Teaching strategies during the education: PBL model (↑, β = 57.48, *p* < .0001) (ref: Traditional teaching model) PBL courses: Professional nursing courses (↑, β = 20.33, *p* < .0001) (ref: None) PBL courses: General education curriculum (↑, β = 30.28, *p* = .0003) (ref: None) length of exposure to PBL 1–20h (↑, β = 97.62, *p* < .0001) (ref: 0h) length of exposure to PBL 21–30h (↑, β = 82.92, *p* < .0001) (ref: 0h) length of exposure to PBL 31–40h (↑, β = 90.23, *p* < .0001) (ref: 0h) |
| 20 | Yoo, 2019, Korea | To examine the relationship between explicit and tacit knowledge sharing and nurses’ CDM, and to test the mediating role of organizational trust. | Cross-sectional survey; structural equation modeling | RNs (n = 210) from four general hospitals | Clinical Decision-Making in Nursing Scale | Explicit knowledge sharing (direct, *b* = 0.31; *p* = .04) Organizational Trust (direct, *b* = 2.82; *p* = .02). Tacit knowledge sharing had no direct effect (*b* = 0.08; *p* = .54) but had an indirect positive effect on CDM abilities through trust (*b* = 0.13; *p* = .04). |
| 21 | ^*^Zuriguel-Pérez, 2018, Spain | To analyze the levels of critical thinking among nurse managers and registered nurses and to explore the association between these levels and socio-demographic and occupational factors. | Cross-sectional survey, multivariate logistic regression | Nurse managers (n = 44) from tertiary hospital | Nursing Critical Thinking in Clinical Practice Questionnaire | Age: 35–46 (OR = 7.62; *p* = .00) Age: 46–52 (OR = 14.48; *p* = .00) Age: 52–65 (OR = 17.67; *p* = .00) Educational level: Master’s degree (OR = 2.32; *p* = .05) Shift schedule/part-time/full-time: Night shift (12h) full-time (OR = 0.34; *p* = .02) Shift schedule/part-time/full-time: Day shift (12h) full-time (OR = 0.01; *p* = .00) |
| 22 | Alaseeri, 2021, Saudi Arabia | To explore the factors that influence nurses’ clinical and managerial decision-making processes (DMPs), and to illustrate how these factors facilitate or inhibit nurses’ ability. | Descriptive qualitative study, thematic analysis | RNs and nurse managers n = 52  from two ministries of health hospitals | Focus group interviews | Theme 1. The Influence of Personal Factors on the Nursing Decision-Making Process  Nurse’s physical and psychological status: sick or not feeling, under pressures and stress  Nurse’s communication skills: language barriers  Nurse’s values, beliefs and cultural awareness  Patients’ different traditional cultures and religious beliefs  Previous experience  Sociocultural norms and Islamic laws  Freedom and authority  Theme 2. The influence of organizational factors effect on decision-making process  Workload: high patient ratio, doing non-nursing jobs, Distractions and interruptions  Availability of resources (human and non-human)  Utilizing technology for patient records and treatment choices  Support and correction from the nursing director or supervisors and hospital administration  Support from expert nurses  Consistence in the application of policies and procedures and the availability of clinical guidelines  floating system within the organization  Noncompliance with hospital policies and rules  Continuous monitoring programs & nursing competency programs |
| 23 | Daemers, 2017, Netherlands | To explore the factors that influence the CDM of independently practicing primary care midwives, focus on how personal, interpersonal, organizational, and contextual elements interact with the evidence-based medicine model. | Descriptive qualitative study, data analyzed by thematic analysis | Primary care midwives (n = 11)  from midwifery practices | In-depth interviews incorporating vignettes and the think aloud method | Theme 1: The pregnant woman as a whole person  Patient physical information  Disadvantaged social environment, low intellectual abilities, hypochondriac, patients with anxiety disorders  Preferences and needs of patients  Theme 2: Sources of knowledge  Gathered knowledge from education and preceptors  Updating knowledge through various methods: reading journals, attending educational programs, using the internet, consulting colleagues, and participating in working groups  Adherence to the guidelines  Perinatal audits  Theme 3: The midwife as a whole person  Attitude to physiology  Attitude to woman-centeredness  Attitude to shared decision-making  Experience and intuition  Attitude towards collaboration (e.g., constructive dialogue or conflict avoidance)  Personal circumstances  Theme 4: Collaboration between maternity care professionals  Making general agreements or discussing individual cases between maternity care professionals  Cooperation with obstetricians  Theme 5: Organization of care  Legislation regarding the working conditions  Quality of medical facilities, financial considerations |
| 24 | Dresser, 2023, USA | To describe how medical surgical nurses perceive the factors that influence their clinical judgment, specifically how they notice, interpret, and respond when caring for deteriorating patients, in order to inform systems and practice improvements for patient safety. | Descriptive qualitative study, thematic analysis | Medical-surgical RNs n = 20  from an academic medical center | Individual semi-structured telephone interviews | Overarching Theme: Nurses’ keen sense of responsibility  Theme 1. Themes related to noticing  (a) Importance of patient assessment: Changes in patients’ deterioration, physically checking on patients  (b) Experience matters: Experience fosters knowledge, gut feelings  (c) Lots of small points where the system can fail: Heavy workload, high nurse to patient ratios, complexity of patient care, available resources, and communication at patient handoff  Theme 2. Themes related to interpreting  (a) Making sense of the data: Availability of information and the presence of a more experienced nurse  (b)Something doesn't go together: Nurses' ability to interpret a situation  Theme 3. Themes related to responding  (a) Caught in the middle: physician conﬂict when calling, communicating, and engaging with physicians  Responding:  a) Caught in the middle: physician conflict when calling, communicating, and engaging with physicians  b) Culture of teamwork: Nursing support, relationship with rapid response teams  c) Increased nursing workload: Time pressures, heavy patient loads, lack of a nurse technician, high acuity patients, juggling multiple patient demands, admissions and discharges, and operational failures related to processes or missing equipment |
| 25 | Lean Keng, 2017, Jordan | To examine the association between critical care nurses’ cognitive bias, decision-making styles (rational and experiential), and leadership styles, and to explore influencing factors through mixed methods. | Mixed methods, simple linear regression and grounded theory analysis | [Quantitative]  Critical care unit RNs (n = 96) | [Quantitative]  Cognitive-Experiential Self-Theory Scale | [Quantitative]  **Rational ability:**  Cognitive bias (↑, b = 0.873, *p* < .001)  Task-oriented leadership style (↑, b = 0.101, *p* = .007)  **Experientiality:** No significant influencing factors found |
|  |  |  |  | [Qualitative]  Critical care unit RNs (n = 20)  from general hospital | [Qualitative]  In-depth interview | [Qualitative]  (a) Being competent: High level of practice competence  (b) Organizational structures: Supportive working environment, Supportive learning environment  (c) Feeling self‑confident: Feeling self‑confident  (d) Being supported: hospital protocol, linked closely to organizational structures |

Note = RNs: Registered Nurses, NP: Nurse Practitioner, ICU: Intensive Care Unit, OPD: Outpatient Department, CTD: Critical Thinking Disposition, CDM: Clinical Decision-Making, DMS: Decision-Making Strategies, PBL: Problem-Based Learning, aOR: Adjusted Odds Ratio, OR: Odds Ratio, β: Beta coefficient, *b*: Regression coefficient, *p*: probability value, *: Participant groups included in the study but not assessed for influencing factors were excluded from the table.
